# *N*-glycan Remodeling Using Mannosidase Inhibitors to Increase High-mannose Glycans on Acid α-Glucosidase in Transgenic Rice Cell Cultures

**DOI:** 10.1038/s41598-018-34438-z

**Published:** 2018-10-31

**Authors:** Hong-Yeol Choi, Heajin Park, Jong Kwang Hong, Sun-Dal Kim, Jun-Young Kwon, SeungKwan You, Jonghye Do, Dong-Yup Lee, Ha Hyung Kim, Dong-Il Kim

**Affiliations:** 1 Department of Biological Engineering, Inha University, 100 Inha-ro, Nam-gu, Incheon, 22212 Republic of Korea; 2 Biotherapeutics and Glycomics Laboratory, College of Pharmacy, Chung-Ang University, 84 Heukseok-ro, Dongjak-gu, Seoul, 06944 Republic of Korea; 3 Bioprocessing Technology Institute, Agency for Science, Technology and Research (A*STAR), 20 Biopolis Way, #06-01, Singapore, 138668 Singapore; 4 School of Chemical Engineering, Sungkyunkwan University, 2066 Seobu-ro, Jangan-gu, Suwon, Gyeonggi-do 16419 Republic of Korea

## Abstract

Glycoengineering of plant expression systems is a prerequisite for the production of biopharmaceuticals that are compatible with animal-derived glycoproteins. Large amounts of high-mannose glycans such as Man_7_GlcNAc_2_, Man_8_GlcNAc_2_, and Man_9_GlcNAc_2_ (Man7/8/9), which can be favorably modified by chemical conjugation of mannose-6-phosphate, are desirable for lysosomal enzyme targeting. This study proposed a rice cell-based glycoengineering strategy using two different mannosidase inhibitors, kifunensine (KIF) and swainsonine (SWA), to increase Man7/8/9 glycoforms of recombinant human acid α-glucosidase (rhGAA), which is a therapeutic enzyme for Pompe disease. Response surface methodology was used to investigate the effects of the mannosidase inhibitors and to evaluate the synergistic effect of glycoengineering on rhGAA. Both inhibitors suppressed formation of plant-specific complex and paucimannose type *N*-glycans. SWA increased hybrid type glycans while KIF significantly increased Man7/8/9. Interestingly, the combination of KIF and SWA more effectively enhanced synthesis of Man7/8/9, especially Man9, than KIF alone. These changes show that SWA in combination with KIF more efficiently inhibited ER α-mannosidase II, resulting in a synergistic effect on synthesis of Man7/8/9. In conclusion, combined KIF and SWA treatment in rice cell culture media can be an effective method for the production of rhGAA displaying dominantly Man7/8/9 glycoforms without genetic manipulation of glycosylation.

## Introduction

Increasing worldwide demand for biopharmaceuticals can be satisfied by the development of different platforms for expressing recombinant proteins with flexibility and economic benefits. Plant cell culture systems have gained increasing attention as a next generation recombinant protein expression platform since they have several benefits, including animal-free production, cost-effective bioprocessing, application of current good manufacturing process, and mammalian-like post-translational modifications^1,2^. However, differences between plant and human glycans represent a major challenge to the practical use of plant expression systems. Due to differences in the final steps of the biosynthetic pathway, plant *N*-glycans lack β1,4-galactose, core α1,6-fucose, and sialic acid but contain potentially immunogenic β1,2-xylose and core α1,3-fucose. In addition, plant complex *N*-glycans are often capped with Lewis a epitope [Fucα1- 4(Galβ1-3)GlcNAc-R]^3^. Thus, in order to create safe and effective plant-derived biopharmaceuticals, the plant *N*-glycosylation pathway should be modulated.

A deficiency of acid α-glucosidase (GAA) which hydrolyses glycogen to glucose in the lysosome causes Pompe disease with the accumulation of glycogen in lysosomes. GAA is a glycoprotein of 110 kDa with seven glycosylation sites which is targeted to the by the mannose-6-phosphate (M6P) receptor (M6PR)^4^.

Therefore, to develop therapeutic proteins for lysosomal storage disorders, it is essential to generate terminal M6P glycans on therapeutic proteins for M6PR-mediated pathway^5^, except for Gaucher disease which requires terminal mannose for uptake by the mannose receptor. In the M6PR-mediated pathway, *in vitro* M6P chemical conjugation depends upon the number of terminal mannoses containing α1,2-glycosidic linkages, which are potential phosphorylation sites^6^.

Glycoengineering could modify glycan structures to improve therapeutic function using various strategies such as genetic engineering or use of glycoprocessing inhibitors^7^. To generate high-mannose (HM) glycans, mannosidases or *N*-acetylglucosaminyltransferase I (*gnt1*) must be knocked-down and/or knocked-out in the *N*-glycosylation biosynthetic pathway^8^. One complementary strategy to genetic engineering techniques is the design of culture media, which can be readily implemented into other glycoprotein expression systems^9^. Especially, glycosidase inhibitors acutely suppress specific *N*-glycan processing enzymes in the *N*-glycosylation pathway obtaining targeted glycoforms, and thus there is no need to generate knock-out cell lines for the modulation of *N*-glycan processing^10^. Kifunensine (KIF) was reported to strongly inhibit α-mannosidase I in the ER (ERMI) and/or *cis*-Golgi apparatus (GMI)^11^, and swainsonine (SWA) inhibits α-mannosidase II in the ER (ERMII) and/or *medial*-Golgi (GMII)^12^. Changes in glycan profiling by each inhibitor were reported in mammalian cell cultures^13,14^, but these approaches have not been applied to plant cell culture systems for the production of glycoproteins.

In this study, rice-derived recombinant human acid α-glucosidases (rrhGAA), which are therapeutic enzyme of the Pompe disease requiring M6PR-mediated uptake, were produced in transgenic rice cells. We employed a design of experiment (DoE) approach using response surface methodology to investigate the effects of two inhibitors on HM glycan profiles. To quantify the effects of the inhibitors and evaluate the synergistic effect on glycan profiling of rrhGAA, glycovariants derived from each condition were analyzed by hydrophilic interaction ultra-high-performance liquid chromatography (HILIC-UPLC) with fluorescence detection and matrix-assisted laser desorption/ionization time-of-flight mass spectrometry (MALDI-TOF MS) after 2-aminobenzamide (2AB) labeling. This study noted the effects of two different mannosidase inhibitors, KIF and SWA, on the glycosylation pathway of rice cells and the production of HM glycans without need for cell line development.

## Results

### Cell culture profiles of transgenic rice cells

**Figure 1:**
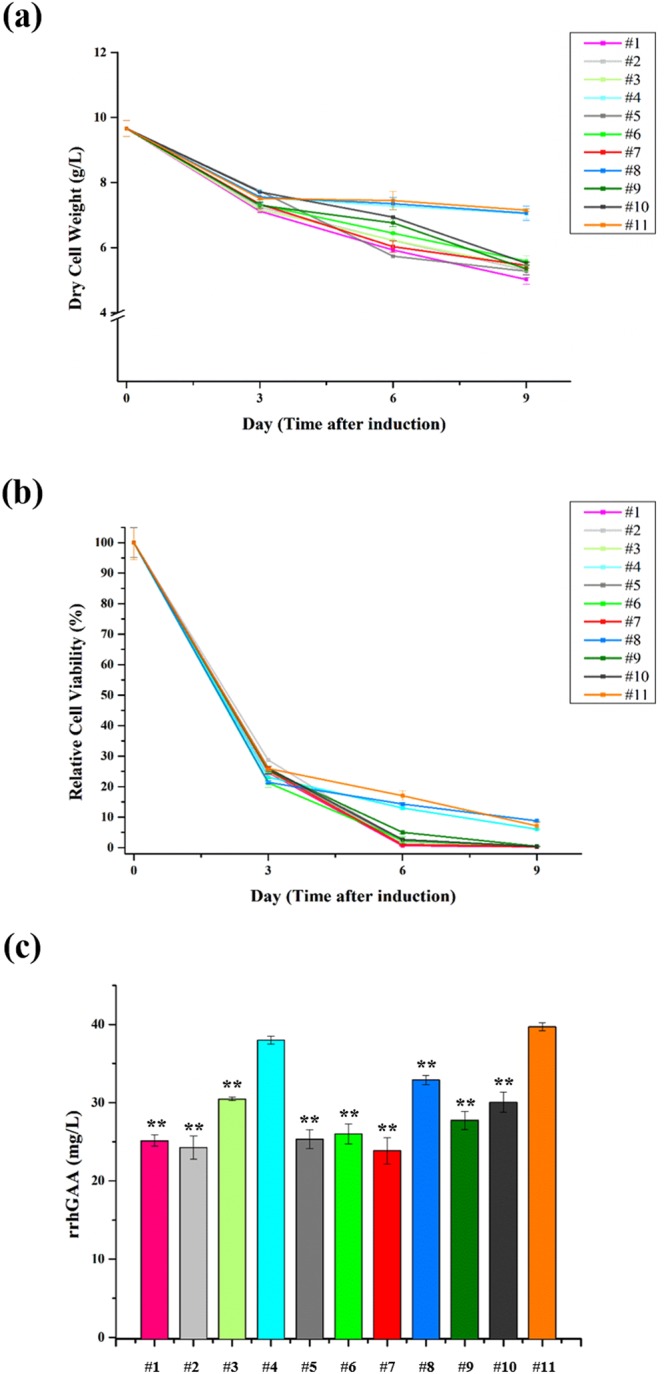
Effects of mannosidase inhibitors on (**a**) cell mass, (**b**) relative cell viability, and (**c**) rrhGAA production on day 9 in rice cell cultures. Cultures were performed in triplicates, and each cell mass and relative cell viability were determined every 3 days after inoculation. Values are represented as the mean ± SD. On day 9, the amounts of rrhGAA in culture media were measured using indirect ELISA. Asterisks indicate the statistical significance (one-way ANOVA) compared to control without mannosidase inhibitors at level **p < 0.01. The coded values of the mannosidase inhibitors are represented in Table 1.

Table 1 represents the coded values in the DoE structure with the concentrations of two mannosidase inhibitors in this study. Compared to the control (#11), KIF alone at 2.5 µM (#1) and 5 µM (#7) resulted in rapid reduction of cell mass (Fig. 1a) and relative cell viability (Fig. 1b) while similar or slightly lower levels were observed from those treated with SWA alone at 5 µM (#8) and 10 µM (#4). The cell mass and relative cell viability profiles of cultures treated with both KIF and SWA (#2, #3, #5, #6, #9 and #10) were similar to those of KIF alone (#1 and #7) regardless of SWA concentration. These results indicate the negative impact of KIF on cell mass and relative cell viability, whereas SWA had little effect. Consistent with the above trends, KIF significantly reduced rrhGAA production while SWA itself slightly decreased rrhGAA production (Fig. 1c and Table S1).

**Table 1:** Coded values of mannosidase inhibitors indicating concentrations of KIF and/or SWA in media.

Condition	#1	#2^a^	#3	#4	#5^a^	#6	#7	#8	#9	#10^a^	#11
Coded values	0	0	0	−1	0	+1	+1	−1	+1	0	−1
	−1	0	+1	+1	0	+1	−1	0	0	0	−1
KIF (µM)	2.5	2.5	2.5	0	2.5	5	5	0	5	2.5	0
SWA (µM)	0	5	10	10	5	10	0	5	5	5	0

All conditions were conducted in triplicates. ^a^Triplicate central point of the face-centered composite design.

### *N*-glycosylation of rrhGAA derived from wild type and *gnt1* knock-out rice cell

**Figure 2:**
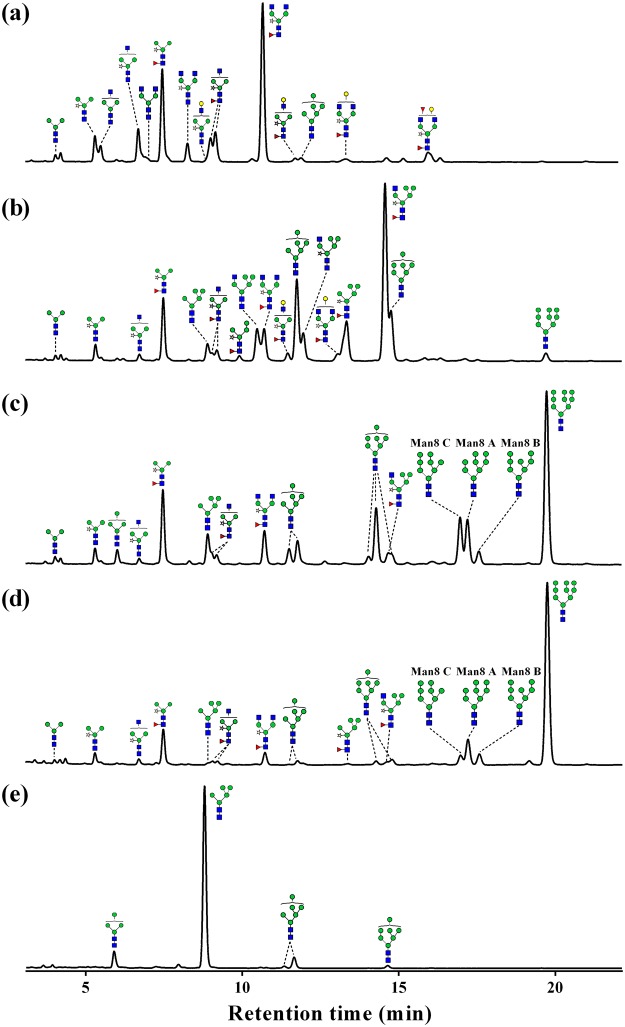
HILIC-UPLC chromatogram of 2AB-labeled glycans of rrhGAA from wild-type rice cell culture treated with SWA and/or KIF and *gnt1* rice cells. (**a**) Control without mannosidase inhibitors, (**b**) 5 µM of SWA, (**c**) 2.5 µM of KIF, (**d**) 5 µM of KIF and 5 µM of SWA, and (**e**) *gnt1*. , *N*-acetylglucosamine; , mannose; , galactose; , fucose; , xylose. Man8 A: Man8 isomer A, Man8 B: Man8 isomer B, Man8 C: Man8 isomer C.

**Figure 3:**
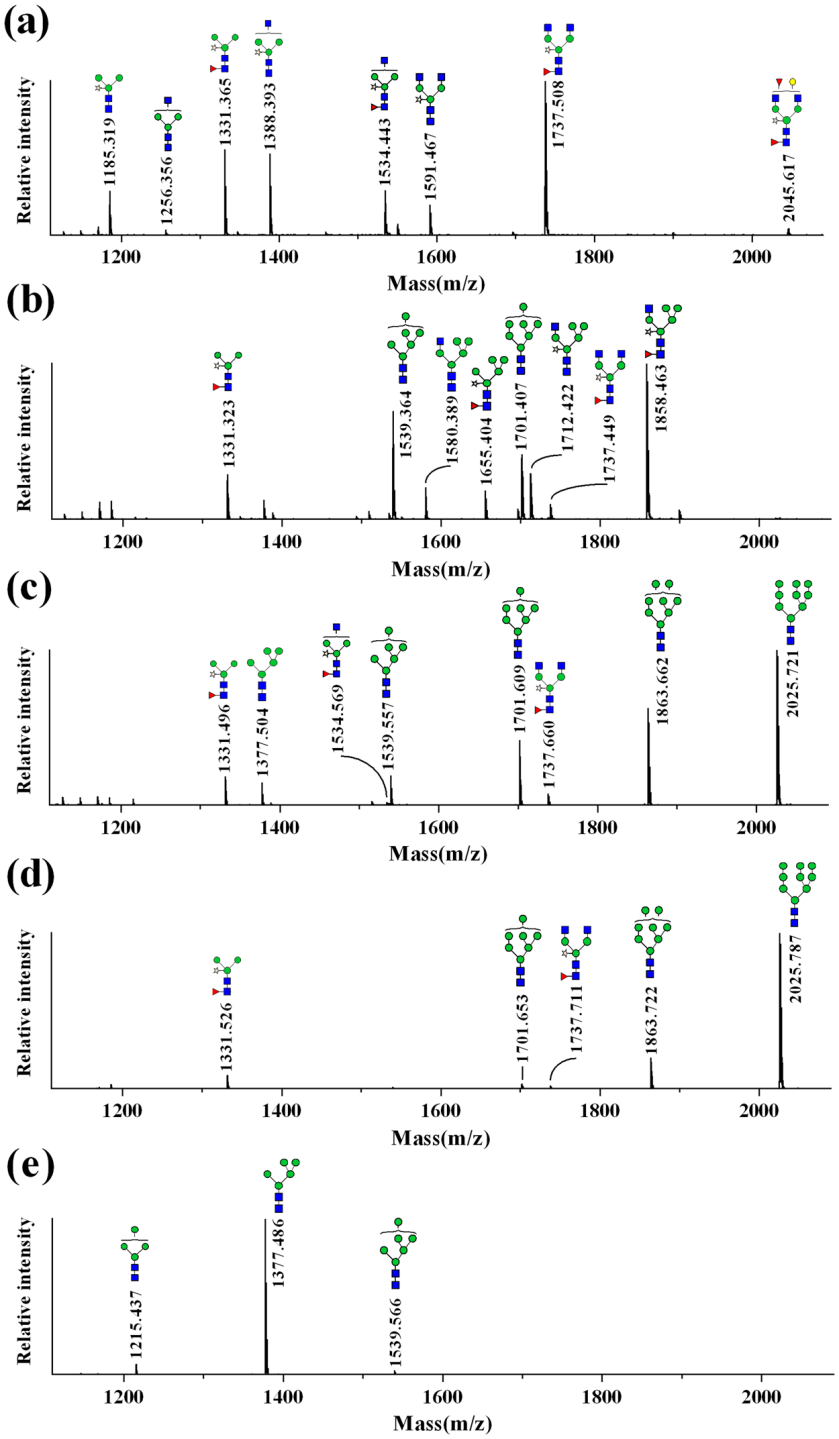
MALDI-TOF mass spectra of 2AB-labeled glycans of rrhGAA from wild-type rice cell culture treated with SWA and/or KIF and *gnt1* rice cells. (**a**) Control without mannosidase inhibitors, (**b**) 5 µM of SWA, (**c**) 2.5 µM of KIF, (**d**) 5 µM of KIF and 5 µM of SWA, and (**e**) *gnt1*. , *N*-acetylglucosamine; , mannose; , galactose; , fucose; , xylose.

The *N*-glycans of rrhGAA produced by wild-type control (#11) consisted of mainly complex type glycans (72.2%), followed by paucimannose type (26.6%) (Figs 2a, 3a and Table S2). Among these, complex type glycan GlcNAc_2_XylFucMan_3_GlcNAc_2_ (GnGnXF^3^) (36.9%) and paucimannose type glycan XylFucMan_3_GlcNAc_2_ (MMXF^3^) (20.2%) comprised more than half of total glycans. In addition to these two major glycoforms, various complex type glycans carrying α1,3-fucose and β1,2-xylose as well as low levels of HM glycan Man_6_GlcNAc_2_ (Man6) (1.1%) were identified, but Man_7/8/9_GlcNAc_2_ (Man7/8/9) glycans and hybrid type glycans were not detected. On the other hand, *gnt1* rrhGAA displayed only HM glycans, including Man5 (84.7%), followed by Man_4_GlcNAc_2_ (Man4) (7.3%), Man6 (5.7%), and Man7 (1.4%) (Figs 2e and 3e).

### Effects of SWA on *N*-glycosylation of rrhGAA derived from wild type

SWA at a concentration of 5 µM (#8) effectively inhibited GMII and converted complex type glycans into hybrid type glycans (51.6%), which were composed mostly of GlcNAc_1_XylFucMan_5_GlcNAc_2_ (Man5GnXF^3^) (30.7%), followed by XylFucMan_5_GlcNAc_2_ (Man5XF^3^) (9.3%), GlcNAc_1_Man_5_GlcNAc_2_ (Man5Gn) (5.9%), GlcNAc_1_XylMan_5_GlcNAc_2_ (Man5GnX) (5.0%), and XylFucMan_4_GlcNAc_2_ (Man4XF^3^) (0.8%) (Figs 2b, 3b and Table S2). In the presence of 5 µM SWA (#8), HM glycans increased from 1.1% at control (#11) to 25.6%, including Man5 (2.5%), Man6 (13.6%), Man7 (7.9%), and Man9 (1.6%), whereas Man4 and Man8 were not observed. Complex type (11.9%) and paucimannose type (11.0%) glycans were significantly reduced by 5 µM SWA treatment. Elevation of SWA concentration (to 10 μM) (#4) increased hybrid type glycans to 61.2% but reduced HM glycans (18.4%). A higher concentration of SWA especially increased hybrid type glycans containing core fucose (Man4XF^3^, Man5XF^3^, and Man5GnXF^3^) but decreased Man5Gn and Man5GnX. The amounts of newly synthesized Man7/8/9 glycans in response to SWA were reduced with increasing SWA concentration from 9.5% at 5 µM SWA (#8) to 4.4% at 10 µM SWA (#4) (Table S1).

### Effects of KIF on *N*-glycosylation of rrhGAA derived from wild type

A specific inhibitor of α-mannosidase I, KIF, efficiently reduced both complex type and paucimannose type glycans, which were abundantly found in wild-type control rrhGAA (#11), while simultaneously increasing HM glycans. KIF at 2.5 µM (#1) increased Man7/8/9 glycans up to 63.8%, of which the most abundant glycan was Man9 (31.9%), followed by Man8 (19.1%) and Man7 (12.8%) (Figs 2c, 3c and Table S2). At 5 µM KIF (#7), Man9 was elevated up to 34.9% while Man7/8 were slightly reduced, resulting in elevation of Man7/8/9 up to 65.4%. In the case of hybrid type glycans, only Man5GnXF^3^ was newly detected up to 2.0% by KIF. These results demonstrate that KIF efficiently inhibited both ERMI and GMI, the target enzyme in the ER and/or *cis*-Golgi, thus increasing Man9 as the most responsive glycan.

### Effects of the combination SWA and KIF on *N*-glycosylation of rrhGAA derived from wild type

A combination of KIF and SWA more effectively suppressed early stage *N*-glycan processing in the ER and Golgi, resulting in elevation of Man9 glycan compared to KIF treatment alone (Figs 2d, 3d and Table S2). While the combination of KIF and SWA led to a moderate increase in the total amount of HM glycans compared to treatment with KIF alone [from 76.8% at 5 µM KIF (#7) to maximum abundance of 81.8% with 5 µM KIF and 10 µM SWA (#6)], there was a significant change in the relative distribution of HM glycans of rrhGAA. Man9 was further elevated by treatment with both KIF and SWA, resulting in similar or lower amounts of Man4/5/6. While Man6/7 were the major HM species up-regulated upon SWA treatment alone, the combination of SWA and KIF primarily up-regulated Man9, leading to relative abundance of the Man9 structure up to 60.4% and simultaneously reduction of Man4/5/6 to 1.5% in the presence of 10 µM SWA and 5 µM KIF (#6). The results show that the highest contents of Man7/8/9 were achieved at the combination of 10 µM SWA and 5 µM KIF (#6) (up to 80.3%) (Table S1). The hybrid type glycans, abundant in SWA-treated rrhGAA, were reduced in the presence of both KIF and SWA.

The changes in Man8 were dependent on the concentration of KIF in the combination groups (#2, #3, #5, #6, #9 and #10). An increasing SWA concentration (from 5 µM to 10 µM) led to a slight decrease in the total amount of Man8 in the presence of 2.5 µM KIF (#2, #5, #10 *vs* #3) while increasing Man8 in combination with 5 µM KIF (#6 *vs* #9). Quantification of Man8 isomers revealed that 2.5 µM KIF (#1) dominantly produced Man8 isomer C (8.4%), a similar or slightly less amount of isomer A (8.2%), and a low amount of isomer B (2.4%) (Fig. 2c and Table S2). This trend in Man8 isomer distribution appeared to be more evident with a higher concentration of KIF at 5 µM (#7), producing 9.3% of Man8 isomer C followed by 7.9% of Man8 isomer A and only 1.8% of Man8 isomer B. Interestingly, additional SWA in KIF-treated cells altered this trend by reducing the relative amount of Man8 isomer C. As a result, Man8 isomer A was the prominent isomer, followed by isomers B and C (Fig. 2d). Depending on the concentrations of SWA and KIF, the total amount of Man8 isomers varied, ranging from 14.6 to 22.3%. However, the trend in Man8 isomers remained unchanged in the presence of SWA and KIF, as Man8 isomer A was the most abundant (8.2~12.3%), followed by isomers B (3.5~6.0%) and C (3.0~4.4%) (Table S2).

### DoE-based approach for analyzing effects of KIF and SWA on the production of rrhGAA with HM glycans in transgenic rice cell cultures

**Figure 4:**
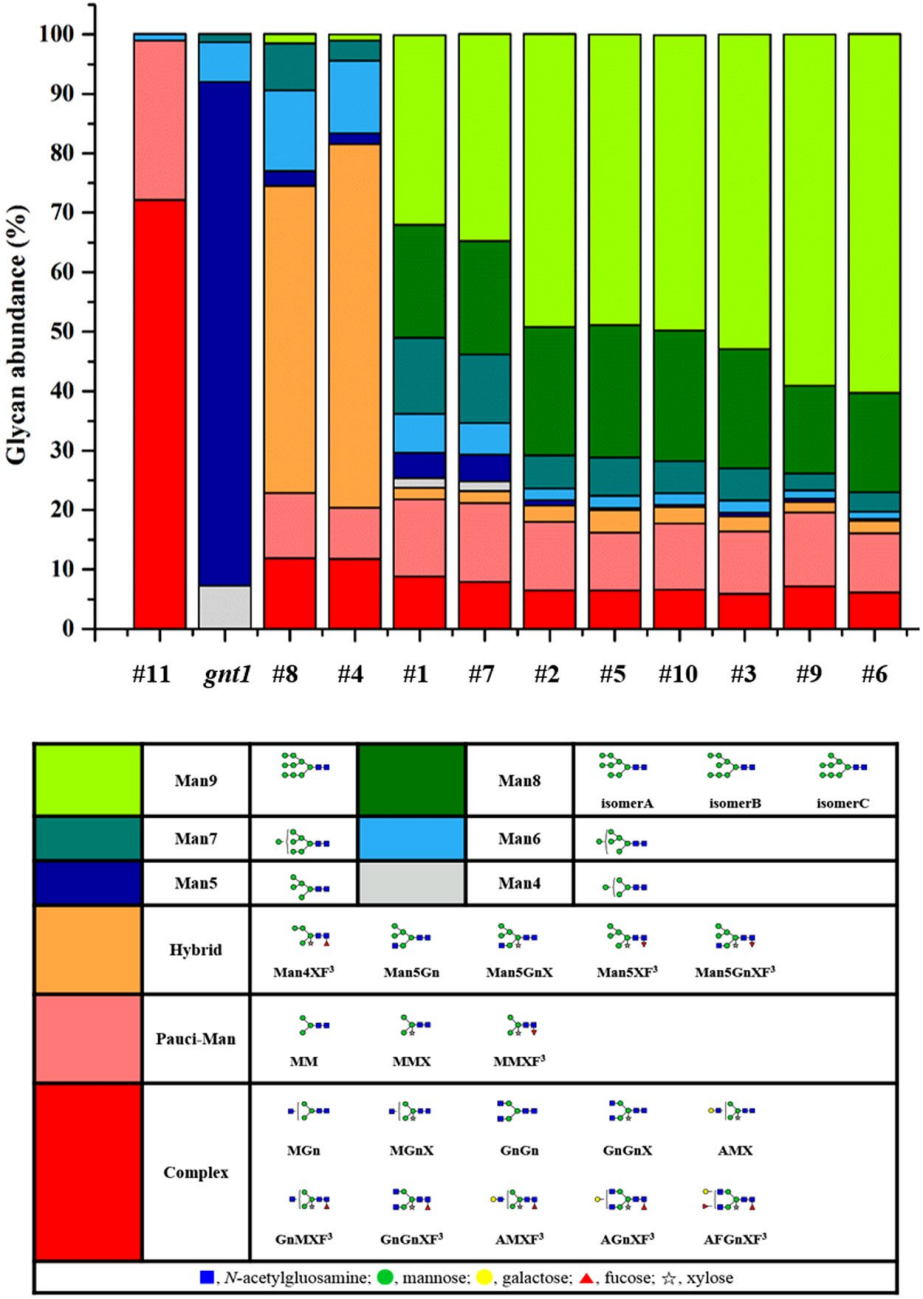
Relative glycan abundance according to the design of experiment using KIF and/or SWA compared to the control culture (#11: wild type rice cells; *gnt1*: *gnt1* knock-out rice cells) without mannosidase inhibitors. Relative glycan abundance of rrhGAA was analyzed by UPLC chromatography after purification. Mean values of glycan abundance (%) are shown (*n* = 3). Different shades of colors represent percentages of each glycovariants, and the corresponding structures of *N*-glycans are indicated below the table.

We hypothesized that a combination of KIF and SWA might increase the proportion of HM glycovariants, especially Man7/8/9 which are desired glycan structures containing potential phosphorylation sites for M6P chemical conjugation in rice cell cultures^10^. As shown in Fig. 4, *N*-glycans derived from SWA alone (#4 and #8) presented relatively low amounts of Man7/8/9, indicating that SWA did not significantly increase Man7/8/9. In contrast, KIF alone at 2.5 µM and 5 µM (#1 and #7) resulted in 63.8% and 65.4% Man7/8/9, respectively, demonstrating that KIF significantly enhanced the proportion of Man7/8/9 in a dose-dependent manner within this range. More interestingly, the other discrete coordinates in the design space, where both KIF and SWA were added, showed further elevation of Man7/8/9 (up to 80.3%), implying an interaction effect of KIF and SWA on Man7/8/9 formation, although SWA itself showed a weak effect on the formation of Man7/8/9 (Table S1). In fact, these contrasting results are derived from the different *N*-glycan processing enzymes inhibited by KIF and SWA; inhibition of ERMI and GMI by KIF in the ER and/or *cis*-Golgi and inhibition of ERMII and GMII by SWA in the ER and/or *medial*-Golgi. The combined treatment could provide a synergistic effect on enhancing formation of Man7/8/9, especially Man9.

**Figure 5:**
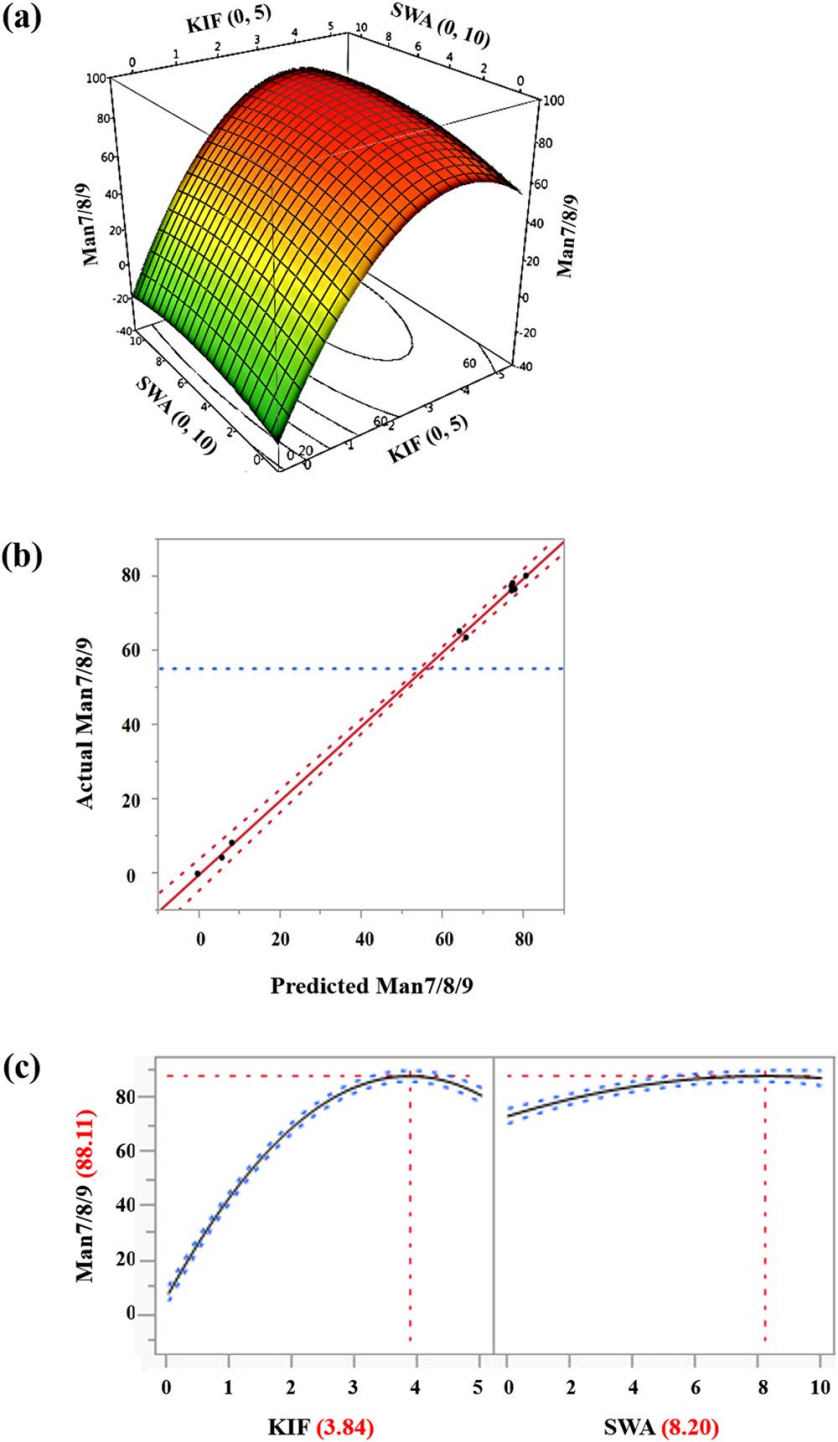
(**a**) Three-dimensional response surface and contour plots of the effects of KIF and SWA on Man7/8/9 contents on day 9. (**b**) Correlation between actual and predicted values of response for Man7/8/9. (**c**) Prediction of the optimal concentration of KIF and SWA. ANOVA is summarized in regression reports (Table S4).

**Figure 6:**
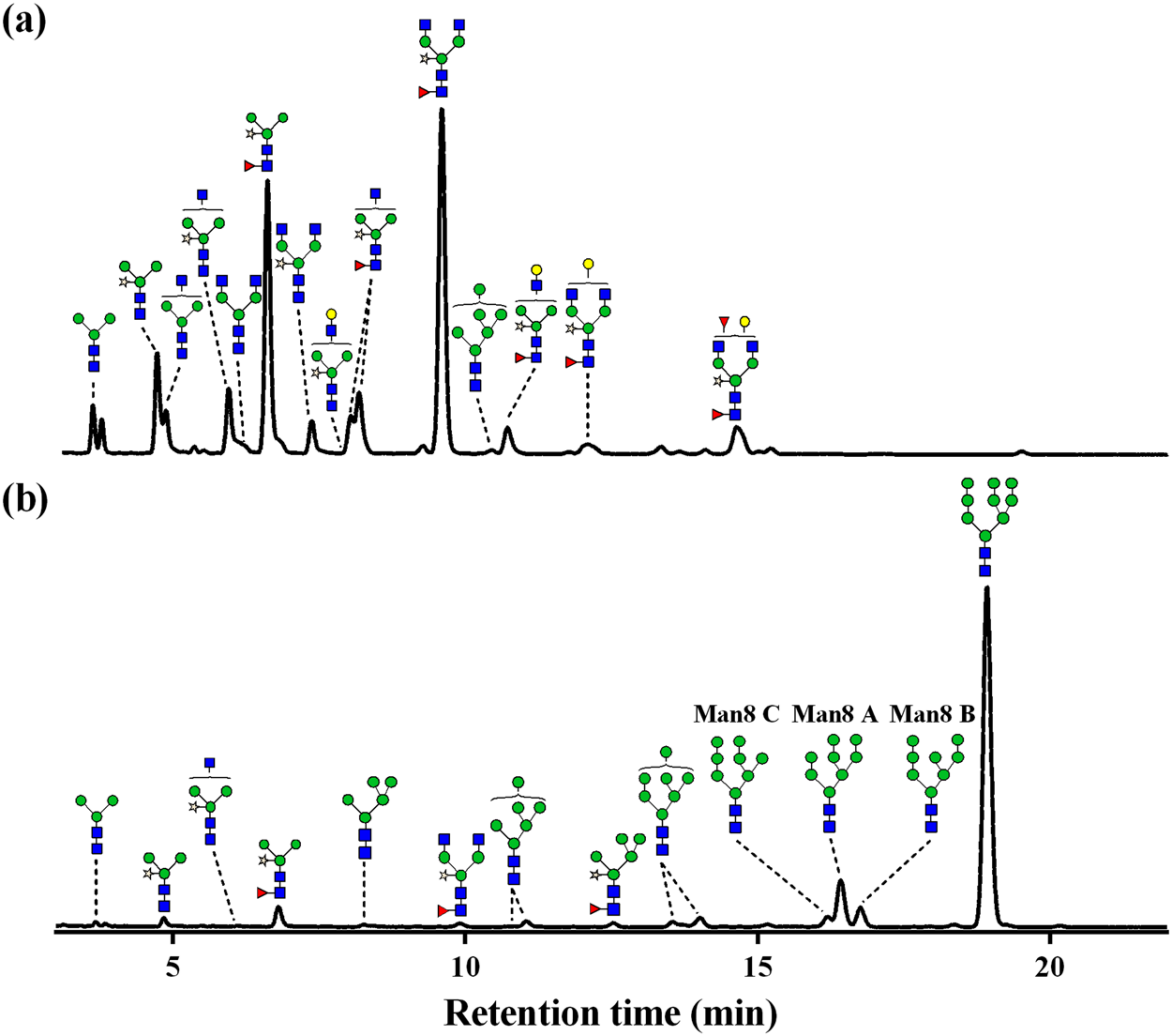
HILIC-UPLC chromatogram of 2AB-labeled glycans of rrhGAA from the rice cell cultures of (**a**) control without mannosidase inhibitors and (**b**) optimal concentration of mannosidase inhibitors (3.84 µM KIF and 8.20 µM SWA) for Man7/8/9 contents. , *N*-acetylglucosamine; , mannose; , galactose; , fucose; , xylose. Man8 A: Man8 isomer A, Man8 B: Man8 isomer B, Man8 C: Man8 isomer C.

Based on the results of the experimental design, specifically Man7/8/9 contents, a response surface model was constructed into the contour plots with input variables (KIF and SWA) and output response (Man7/8/9 contents). These results including contour plot (Fig. 5a) and scaled estimates of main effects on Man7/8/9 (Table S3) revealed that KIF was more influential on Man7/8/9 than SWA. Interestingly, the interaction term (KIF*SWA, p = 0.023) appeared to be statistically significant, indicating a considerable synergistic effect. From the regression model, optimal concentrations of KIF and SWA (3.84 µM KIF and 8.20 µM SWA) achieving the maximum level of Man7/8/9 (88.1 ± 2.0%) were calculated within the experimental ranges (Fig. 5c and Table S4). To validate these optimal values of input and output, additional cultures were conducted with both optimal point (combination of 3.84 µM KIF and 8.20 µM SWA) and control condition (without mannosidase inhibitors) (Fig. 6 and Table S5). While the measured content of Man7/8/9 at optimal point (91.3%) was slightly higher than expected content (88.1 ± 2.0%), the overall glycan profiles were consistent with the expected ones from the regression model, thus providing the statistical confidence of response surface methodology for media optimization. More importantly, purified rrhGAAs in both conditions showed similar levels of *in vitro* enzymatic activity compared with Myozyme (Fig. S1), demonstrating that the treatment of mannosidase inhibitors in rice cell cultures to modulate *N*-glycan structure of rrhGAA may not significantly affect functional activity of rrhGAA.

## Discussion

Herein, we investigated production media using two different mannosidase inhibitors, KIF and SWA, for *N*-glycosylation control in rrhGAA in rice cell cultures. To quantify the effects of the inhibitors, a DoE approach was adopted in this study. Response surface models for Man7/8/9 contents in rrhGAA was established using KIF and SWA as input variables, thus enabling prediction of optimal concentrations of KIF and SWA to achieve the target level of Man7/8/9 with statistical confidence and further understanding of the interactions among the inhibitors behind the relevant mechanism.

Most *N*-glycan in rrhGAA produced in the wild-type control (#11) was fully processed in the Golgi apparatus and vacuole, resulting in abundant plant specific complex type and paucimannose type glycans. KIF treatment conditions (#1 and #7) exhibited large amounts of HM glycans, including primarily Man9 followed by Man8, indicating strong inhibition of ERMI and/or GMI in the *N*-glycosylation pathway. KIF treatment yielded smaller amounts of Man8 isomer B, a product of ERMI, than Man8 isomers A and C, which can be rapidly degraded by GMI in wild-type rice cells^15^. The increased concentration of KIF from 2.5 µM to 5 µM elevated Man9 levels and reduced Man8 isomer B level. There was a minor amount of hybrid type glycans (1.9~2.0%), confirming that KIF can act as a weak inhibitor of GMII^16^. On the other hand, SWA conditions (#4 and #8) resulted in mainly hybrid type glycans and HM glycans composed of Man6 and Man7, possibly due to potent GMII inhibition and minor ERMII inhibition. This study demonstrated for the first time that the combination of KIF and SWA (#2, #3, #5, #6, #9 and #10) strongly inhibited the mannose trimming pathway related to ERMI, GMI, and ERMII in the *N*-glycosylation pathway of rice cells and led to dramatic increases in Man7/8/9 of rrhGAA derived from rice cells (up to 80.3%). The amount of Man9 was most prominently affected among HM glycans in response to the combination of SWA and KIF compared to SWA or KIF treatment alone. As high contents of Man7/8/9 glycans are critical for efficient *in vitro* M6P chemical conjugation to enhance M6PR-mediated uptake pathway^17^, this result is valuable for improving the quality of the rrhGAA producing-rice cell culture process. Interestingly, addition of SWA to rice cell culture in the presence of KIF altered the relative distribution of Man8 isomers. The amount of Man8 isomer C, which is the major structure among Man8 isomers produced by KIF alone (Fig. 2c), decreased while the amount of Man8 isomer A increased (Fig. 2d). Man8 isomer A, the product of endomannosidase, is able to circumvent the glycosylation process inhibited by SWA and KIF^15^. This result provides evidence that SWA could act as a potent ERMII inhibitor only when treated along with KIF, as SWA treatment alone was insufficient to block ERMII. In addition, hybrid type glycans, which were the most abundant glycan species in SWA alone, accounted for only 1.8~3.8% of rrhGAA obtained from the combination of SWA and KIF. It is highly possible that the *N*-glycosylation process was more efficiently arrested at Man8 and Man9 in the ER by the combination of SWA and KIF, suggesting that SWA is not effective in producing hybrid type glycans in the Golgi apparatus. Despite the stronger effect of KIF on HM glycan formation, KIF treatment in rice cell cultures had a negative effect on culture performance parameters such as cell mass, cell viability, and rrhGAA titer. In contrast, SWA did not significantly impair these cell culture profiles or HM glycan formation. Although these combination conditions had a negative effect on cell culture performance, these impacts were not worse compared to KIF alone.

**Figure 7:**
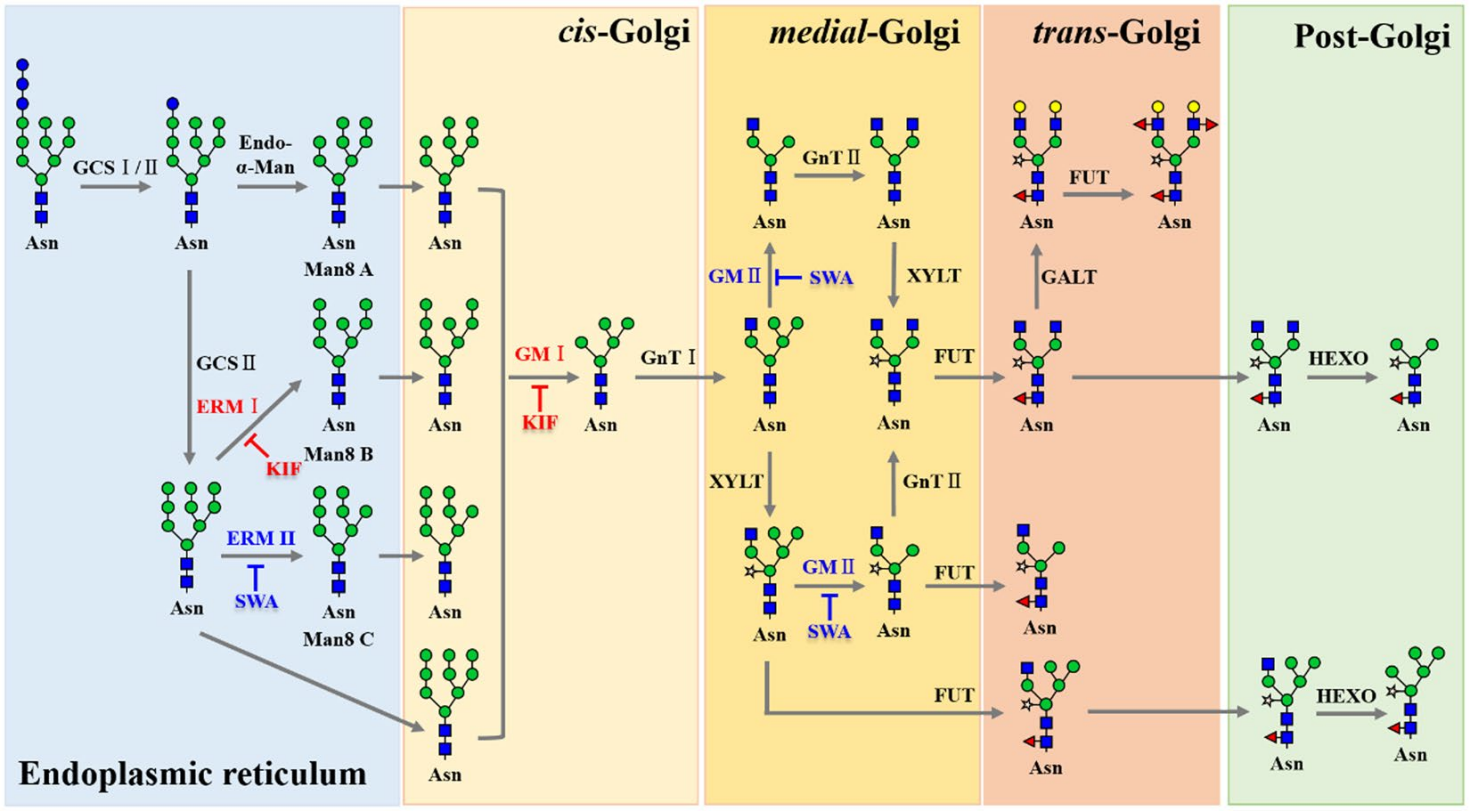
Putative *N*-glycosylation pathway in rice (*O. sativa*) cell and the effects of mannosidase inhibitors. , *N*-acetylglucosamine; , mannose; , galactose; , fucose; , xylose. GCSI/II: α-glucosidases I/II, Endo-α-Man: endomannosidase, ERMI/II: ER α-mannosidase I/II, GMI/II: Golgi α-mannosidase I/ II, GnTI/II: β1,2-*N*-acetylglucosaminyltransferase I/II, FUT: α1,3-fucosyltransferase, XYLT: β1,2-xylosyltransferase, HEXO: β-*N*-acetylhexosaminidases, Asn: *N*-glycosylation site, Man8 A: Man8 isomer A, Man8 B: Man8 isomer B, Man8 C: Man8 isomer C.

We found that the combination of KIF and SWA dramatically increased HM glycan contents on rrhGAA compared to each of the inhibitors alone, which has not been previously reported. Interestingly, this approach successfully eliminated plant-specific glycan variants in rrhGAA, thus overcoming the major drawback of plant cell culture systems for mammalian protein production. Moreover, detailed analysis of glycovariants enabled to understand the *N*-glycosylation pathway in callus-derived suspension rice cells (*O. sativa*), thereby providing more information on recombinant protein development in the rice cell culture platform (Fig. 7). Compared to the genetic manipulation of the rice host cell lines for desired glycan profile of the products requiring considerable time and efforts, this strategy is easily and immediately applicable to the rice cell culture process and thus more feasible in the biopharmaceutical industry. While knock-out approach such as *gnt1* mutant used in this study may be efficient to modulate phenotypes in on/off mode, media optimization approach with specific enzyme inhibitors can offer more rapid and flexible operating space identification depending on the target quality attributes, thus rendering economic benefits in the rice cell culture process development. Further research and engineering of the glycosylation in rice cell culture for mammalian protein production will accelerate rice cell culture systems as an alternative production platform in the biopharmaceutical industry.

## Materials and Methods

### Transgenic rice cell cultures and maintenance

Transgenic wild and *gnt1* knock-out rice (*Oryza sativa* L.) cells producing rrhGAA were donated by Prof. Yang (Chonbuk National University, Republic of Korea). Liquid Chu medium^18^ consisting of 30 g/L of sucrose, 2 mg/L of 2,4-dichlorophenoxyacetic acid, and 0.02 mg/L of kinetin was used for maintenance of rice cells. For the statistical experiments, 10% (w/v) rice cells were inoculated in a 100-mL flask containing 30 mL of Chu medium without sucrose^19^, and suspension cells were cultured at 28 °C and 110 rpm in the dark in a shaking incubator. KIF (Enzo Life Sciences, NY, USA) at 0 to 5 µM and SWA (Enzo Life Sciences) at 0 to 10 µM were supplemented to medium as inhibitors of mannosidases in the *N*-glycosylation pathway. Cell culture profiles were monitored every 3 days for 9 days.

### Experimental designs and statistical analysis

Determination of the concentration ranges of mannosidase inhibitors was carried out based on previous literature^11,20^, and experiments were designed based on the combinatorial structure of the face-centered response model using JMP 11 software (SAS Institute). Design of the experiment along with values of HM glycan contents and titer is given in Table S1. Experiments and sample analysis were carried out in triplicates, and data from cell culture profiles was expressed as the mean of triplicate determinations. Regression analysis by ANOVA was conducted using the data obtained from the experimental design. Data values were considered significant at p < 0.05.

### Cell mass measurement and relative cell viability assay

Suspension-cultured cells were filtered using Whatman No. 1 filter paper (Sigma-Aldrich, St. Louis, MO, USA) under vacuum conditions, and the cells were washed with distilled water followed by drying at 60 °C for 2 days. Cell mass was estimated by measuring dry cell weight. Assay of relative cell viability was performed through measurement of absorbance at 485 nm using triphenyl tetrazolium chloride solution^21^.

### Quantification of rrhGAA

Indirect enzyme-linked immunosorbent assay was used for quantification of rrhGAA in media. As a standard substance, Myozyme^®^ (Genzyme, Cambridge, MA, USA) was diluted to prepare standard solutions ranging from 10 to 100 ng/mL in bicarbonate buffer solution (15 mM Na_2_CO_3_ and 35 mM NaHCO_3_; pH 9.6). Culture media containing rrhGAA were diluted from 1:1000 to 1:2000 in bicarbonate buffer. Standard and samples were loaded in a 96-well microplate (Sigma-Aldrich) and coated overnight at 4 °C. Custom rabbit anti-GAA polyclonal antibody (Abfrontier, Seoul, Republic of Korea) was used as a primary antibody and loaded with 100 µL/well of the 1:2000 dilution. Peroxidase-labeled goat anti-rabbit IgG (Thermo Fisher Scientific, Waltham, MA, USA) was used to detect primary antibody and loaded with 100 µL/well of the 1:5000 dilution. Development step was performed using 100 µL/well of ultra tetramethylbenzidine (Thermo Fisher Scientific) and then stopped by 2 M sulfuric acid. The absorbance was measured by microplate reader (Thermo Fisher Scientific) at 405 nm.

### Purification of rrhGAA

Suspension cultured media were filtered using a 0.22 µm Millipore Express^®^ PLUS membrane filter (Millipore, Volketswil, Switzerland). His-tagged rrhGAAs in the filtrate were purified using Ni-NTA Superflow Cartridges (Qiagen, Valencia, CA, USA) according to the manufacturer’s protocols. Confirmation of purified rrhGAA was performed by SDS-PAGE analysis (Fig. S2).

### *N*-glycan release and labeling

Enzymatic deglycosylation was performed according to a slightly modified method of Hwang *et al*.^22^ rrhGAA samples were treated with trypsin (Sigma-Aldrich) and chymotrypsin (Sigma-Aldrich) in 10 mM Tris–HCl buffer, pH 8.0, at 37 °C for 18 h. *N*-glycans were liberated by glycoamidase A (Roche Diagnostics, Mannheim, Germany) in citrate-phosphate buffer, pH 5.0, at 37 °C for 18 h and purified using a graphitized carbon cartridge (Alltech, Deerfield, IL, USA)^23^. The released *N*-glycans were fluorescently derivatized via reductive amination with 2AB (Sigma-Aldrich) according to the method described by Hwang *et al*. method^22^. The excess labeling reagent was removed by solid phase extraction packed with a microcrystalline cellulose (Sigma-Aldrich). The purified glycans were lyophilized and stored at −20 °C.

### HILIC-UPLC analysis of *N*-glycans

*N*-glycan profiling was accomplished using a Waters Acquity H-Class UPLC fluorescence detector (excitation = 330 nm, emission = 420 nm) (Waters, Milford, MA, USA). Separation of 2AB-labeled *N*-glycans was carried on a Waters Acquity UPLC BEH Glycan column (1.7 µm, 2.1 × 100 mm, Waters) at 40 °C using 50 mM ammonium formate (pH 4.4) as Solvent A and acetonitrile as Solvent B. The following gradient conditions were applied: t = 0 min, 25% solvent A (0.5 mL/min); t = 46.5, 50% solvent A (0.5 mL/min); t = 48, 100% solvent A (0.25 mL/min); t = 49, 100% solvent A (0.25 mL/min); t = 50, 25% solvent A (0.5 mL/min); t = 63, 25% solvent A (0.5 mL/min). The peaks were calibrated by running an external standard of 2AB-labeled dextran ladder (2AB–glucose homopolymer, Ludger Ltd) and the preliminary identification were performed using GlycoStore database (www.glycostore.org). The individual structures of *N*-glycans were confirmed by HILIC separation coupled to electrospray ionization mass spectrometry (Thermo Scientific, Waltham, MA, USA) (data not shown).

### MALDI-TOF MS analysis of *N*-glycans

MALDI-TOF MS was performed with the Ultraflex III system (Bruker Daltonics, Bremen, Germany) controlled by Flex Control 3.0 (Bruker Daltonics). The mass spectra were measured with reflectron positive ionization mode within 500–3,000 Da. For all analyses, 2,5-dihydroxybenzoic acid (Sigma-Aldrich) was used as the matrix at a concentration of 10 mg/mL in acetonitrile/water (50:50, v/v). Dried 2AB-labeled glycans were dissolved in water, and each sample solution (1 μL) was mixed with appropriate matrix solution (1 μL). These samples were then loaded onto the stainless steel target and dried at room temperature.

## Electronic supplementary material

Supporting Data
