# Supplementary material for: N-glycan Remodeling Using Mannosidase Inhibitors to Increase High-mannose Glycans on Acid α-Glucosidase in Transgenic Rice Cell Cultures
Source: Sci Rep. 2018 Oct 31;8:16130. doi: 10.1038/s41598-018-34438-z (PMC6208381; doi:10.1038/s41598-018-34438-z)
Supplement: Supplementary file 1 — Supporting Data [file 41598_2018_34438_MOESM1_ESM.docx]

***N*-glycan Remodeling Using Mannosidase Inhibitors to Increase High-mannose Glycans on Acid α-Glucosidase in Transgenic Rice Cell Cultures**

Hong-Yeol Choi^1,†^, Heajin Park^2,†^, Jong Kwang Hong^3^, Sun-Dal Kim^1^, Jun-Young Kwon^1^, SeungKwan You^2^, Jonghye Do^2^, Dong-Yup Lee^3,4^, Ha Hyung Kim^2,*^, Dong-Il Kim^1,*^

^1^Department of Biological Engineering, Inha University, 100 Inha-ro, Nam-gu, Incheon 22212, Republic of Korea

^2^Biotherapeutics and Glycomics Laboratory, College of Pharmacy, Chung-Ang University, 84 Heukseok-ro, Dongjak-gu, Seoul 06944, Republic of Korea

^3^Bioprocessing Technology Institute, Agency for Science, Technology and Research (A*STAR), 20 Biopolis Way, #06-01, Singapore 138668, Singapore

^4^School of Chemical Engineering, Sungkyunkwan University, 2066 Seobu-ro, Jangan-gu, Suwon, Gyeonggi-do 16419, Republic of Korea

^†^ These authors contributed equally to this article.

* Correspondence (Tel +82-32-860-7515; fax +82-32-872-4046; email kimdi@inha.ac.kr (D.-I. Kim) and Tel +82-2-820-5612; fax +82-2-823-5612; email hahyung@cau.ac.kr (H.H. Kim))

Heajin Park’s present address: Department of Chemistry, University of Alberta, Edmonton, Alberta T6G 2G2, Canada

Supporting Data

**Table S1.** Structure of experimental design by face-centered composite model and experimental results of rrhGAA production and Man7/8/9 contents. Data were expressed as the mean of triplicate determinations

| Condition order | Sample  label | KIF | SWA | rrhGAA  (mg/L) | Relative abundance of Man7/8/9 (%Area) |
| --- | --- | --- | --- | --- | --- |
| 1 | #1 | 0 | -1 | 25.15 ± 1.00 | 63.8 ± 0.1 |
| 2 | #2 | 0 | 0 | 24.27 ± 2.08 | 76.4 ± 0.1 |
| 3 | #3 | 0 | +1 | 30.48 ± 0.32 | 78.4 ± 0.5 |
| 4 | #4 | -1 | +1 | 37.99 ± 0.70 | 4.4 ± 0.0 |
| 5 | #5 | 0 | 0 | 25.33 ± 1.68 | 77.6 ± 0.1 |
| 6 | #6 | +1 | +1 | 26.02 ± 1.78 | 80.3 ± 0.3 |
| 7 | #7 | +1 | -1 | 23.85 ± 2.36 | 65.4 ± 0.0 |
| 8 | #8 | -1 | 0 | 32.90 ± 0.85 | 9.5 ± 0.1 |
| 9 | #9 | +1 | 0 | 27.73 ± 1.14 | 76.7 ± 0.6 |
| 10 | #10 | 0 | 0 | 30.03 ± 1.81 | 77.3 ± 0.3 |
| 11 | #11 | -1 | -1 | 39.70 ± 0.73 | 0.00 |

**Table S2.** Relative abundance of glycans from rrhGAA collected at different concentrations of SWA and/or KIF

| Sample label | | #11 | #8 | #4 | #1 | #7 | #2 | #5 | #10 | #3 | #9 | #6 |  |
| --- | --- | --- | --- | --- | --- | --- | --- | --- | --- | --- | --- | --- | --- |
| KIF (μM) | | 0 | 0 | 0 | 2.5 | 5 | 2.5 | 2.5 | 2.5 | 2.5 | 5 | 5 |  |
| SWA (μM) | | 0 | 5 | 10 | 0 | 0 | 5 | 5 | 5 | 10 | 5 | 10 |  |
| *N*-glycan structure | | Relative abundance (%)^a^ | | | | | | | | | | | |
| Paucimannose type glycan | |  |  |  |  |  |  |  |  |  |  |  |  |
| MM | 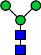 | 1.1 ± 0.1 | 0.4 ± 0.0 | - | 0.8 ± 0.0 | 1.0 ± 0.0 | 0.4 ± 0.0 | 0.4 ± 0.1 | 0.6 ± 0.1 | 0.5 ± 0.0 | 0.6 ± 0.0 | 0.3 ± 0.0 |  |
| MMX | 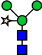 | 5.5 ± 0.1 | 1.9 ± 0.0 | 1.0 ± 0.0 | 1.9 ± 0.0 | 2.1 ± 0.0 | 1.6 ± 0.0 | 1.4 ± 0.0 | 1.7 ± 0.0 | 2.2 ± 0.0 | 2.6 ± 0.1 | 1.5 ± 0.0 |  |
| MMXF^3^ | 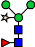 | 20.2 ± 0.2 | 8.6 ± 0.0 | 7.7 ± 0.1 | 10.3 ± 0.1 | 10.3 ± 0.0 | 9.4 ± 0.1 | 7.9 ± 0.1 | 8.9 ± 0.0 | 7.8 ± 0.0 | 9.2 ± 0.4 | 8.2 ± 0.1 |  |
| Complex type glycan | |  |  |  |  |  |  |  |  |  |  |  |  |
| MGn | 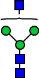 | 3.3 ± 0.0 | - | - | - | - | - | - | - | - | - | - |  |
| MGnX | 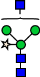 | 7.6 ± 0.2 | 0.8 ± 0.0 | - | 0.6 ± 0.0 | 0.8 ± 0.1 | 0.5 ± 0.0 | 0.5 ± 0.0 | 0.4 ± 0.0 | 0.6 ± 0.0 | 1.4 ± 0.1 | 0.5 ± 0.0 |  |
| GnGn | 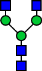 | 1.5 ± 0.1 | - | - | - | - | - | - | - | - | - | - |  |
| GnGnX | 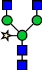 | 4.0 ± 0.1 | - | - | - | - | - | - | - | - | - | - |  |
| AMX | 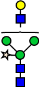 | 0.7 ± 0.2 | - | - | - | - | - | - | - | - | - | - |  |
| GnMXF^3^ | 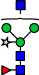 | 12.6 ± 0.2 | 2.7 ± 0.0 | 2.7 ± 0.0 | 3.0 ± 0.1 | 2.5 ± 0.0 | 2.2 ± 0.1 | 2.2 ± 0.1 | 2.2 ± 0.1 | 1.9 ± 0.1 | 2.1 ± 0.1 | 2.0 ± 0.1 |  |
| GnGnXF^3^ | 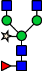 | 36.9 ± 0.3 | 5.9 ± 0.1 | 5.7 ± 0.2 | 5.2 ± 0.0 | 4.5 ± 0.2 | 3.8 ± 0.2 | 3.8 ± 0.2 | 4.0 ± 0.0 | 3.3 ± 0.1 | 3.7 ± 0.1 | 3.6 ± 0.1 |  |
| AMXF^3^ | 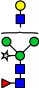 | 0.9 ± 0.1 | 1.2 ± 0.0 | 1.7 ± 0.1 | - | - | - | - | - | - | - | - |  |
| AGnXF^3^ | 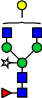 | 1.1 ± 0.1 | 1.2 ± 0.0 | 1.6 ± 0.0 | - | - | - | - | - | - | - | - |  |
| AFGnXF^3^ | 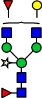 | 3.7 ± 0.2 | - | - | - | - | - | - | - | - | - | - |  |
| Table S2 *(Continued)* | | | | | | | | | | | | | |
| Sample label | | #11 | #8 | #4 | #1 | #7 | #2 | #5 | #10 | #3 | #9 | #6 |  |
| KIF (μM) | | 0 | 0 | 0 | 2.5 | 5 | 2.5 | 2.5 | 2.5 | 2.5 | 5 | 5 |  |
| SWA (μM) | | 0 | 5 | 10 | 0 | 0 | 5 | 5 | 5 | 10 | 5 | 10 |  |
| *N*-glycan structure | | Relative abundance (%)^a^ | | | | | | | | | | | |
| Hybrid type glycan | |  |  |  |  |  |  |  |  |  |  |  |  |
| Man4XF^3^ | 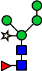 | - | 0.8 ± 0.0 | 1.1 ± 0.0 | - | - | - | - | - | - | - | - |  |
| Man5Gn | 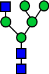 | - | 5.9 ± 0.0 | 3.6 ± 0.0 | - | - | - | - | - | - | - | - |  |
| Man5GnX | 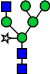 | - | 5.0 ± 0.0 | 3.3 ± 0.1 | - | - | - | - | - | - | - | - |  |
| Man5XF^3^ | 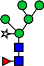 | - | 9.3 ± 0.1 | 11.9 ± 0.1 | - | - | 1.0 ± 0.0 | 1.4 ± 0.1 | 1.0 ± 0.0 | 0.8 ± 0.0 | 0.6 ± 0.0 | 0.7 ± 0.1 |  |
| Man5GnXF^3^ | 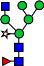 | - | 30.7 ± 0.1 | 41.3 ± 0.2 | 1.9 ± 0.0 | 2.0 ± 0.0 | 1.8 ± 0.3 | 2.4 ± 0.1 | 1.7 ± 0.1 | 1.7 ± 0.1 | 1.2 ± 0.0 | 1.4 ± 0.0 |  |
| High-mannose type glycan | |  |  |  |  |  |  |  |  |  |  |  |  |
| Man4 | 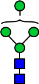 | - | - | - | 1.7 ± 0.0 | 1.6 ± 0.1 | - | - | - | - | - | - |  |
| Man5 | 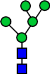 | - | 2.5 ± 0.0 | 1.8 ± 0.0 | 4.2 ± 0.0 | 4.5 ± 0.0 | 0.9 ± 0.0 | 0.3 ± 0.0 | 0.4 ± 0.0 | 0.6 ± 0.0 | 0.5 ± 0.0 | 0.3 ± 0.0 |  |
| Man6 | 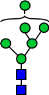 | 1.1 ± 0.1 | 13.6 ± 0.1 | 12.2 ± 0.0 | 6.6 ± 0.0 | 5.4 ± 0.2 | 1.9 ± 0.0 | 2.1 ± 0.0 | 1.8 ± 0.0 | 2.1 ± 0.3 | 1.5 ± 0.0 | 1.1 ± 0.0 |  |
| Man7 | 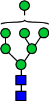 | - | 7.9 ± 0.1 | 3.4 ± 0.1 | 12.8 ± 0.1 | 11.5 ± 0.0 | 5.6 ± 0.0 | 6.3 ± 0.5 | 5.5 ± 0.0 | 5.4 ± 0.2 | 3.0 ± 0.2 | 3.3 ± 0.1 |  |
| Man8 isomerC | 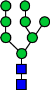 | - | - | - | 8.4 ± 0.1 | 9.3 ± 0.1 | 4.4 ± 0.0 | 4.0 ± 0.0 | 4.3 ± 0.0 | 3.6 ± 0.1 | 3.0 ± 0.0 | 3.5 ± 0.0 |  |
| Man8 isomerA | 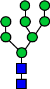 | - | - | - | 8.2 ± 0.0 | 7.9 ± 0.1 | 11.5 ± 0.0 | 12.3 ± 0.1 | 12.0 ± 0.1 | 11.3 ± 0.2 | 8.2 ± 0.0 | 9.1 ± 0.0 |  |
| Man8 isomerB | 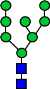 | - | - | - | 2.4 ± 0.0 | 1.8 ± 0.0 | 5.7 ± 0.0 | 6.0 ± 0.0 | 5.7 ± 0.1 | 5.2 ± 0.2 | 3.5 ± 0.0 | 4.1 ± 0.0 |  |
| Man9 | 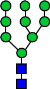 | - | 1.6 ± 0.0 | 1.0 ± 0.0 | 31.9 ± 0.1 | 34.9 ± 0.2 | 49.3 ± 0.1 | 49.3 ± 0.5 | 49.7 ± 0.0 | 52.9 ± 0.2 | 59.1 ± 0.4 | 60.4 ± 0.3 |  |

^a^Values are the average of triplicates instrument runs from the same preparation.

-, not detected; ■, *N*-acetylglucosamine; ●, mannose; ●, galactose; ▲, fucose; ☆, xylose.

**Table S3.** Scaled estimates of main and interaction effects of KIF and SWA on Man7/8/9 abundance (%)

| Term | Scaled Estimate | Standard Error | t Ratio | Prob (P) > │t│ |
| --- | --- | --- | --- | --- |
| Intercept | 76.99 | 0.84 | 91.79 | < 0.0001 |
| KIF (-1, +1) | 34.76 | 0.67 | 52.07 | < 0.0001 |
| SWA (-1, +1) | 5.67 | 0.67 | 8.49 | 0.0004 |
| KIF*SWA | 2.65 | 0.82 | 3.24 | 0.0229 |
| KIF*KIF | -33.78 | 1.03 | -32.88 | < 0.0001 |
| SWA*SWA | -5.76 | 1.03 | -5.60 | 0.0025 |

**Table S4.** Summary of ANOVA for Man7/8/9 regression model

| Source | SS | DF | MS | *F*-value | Prob (*P*) > *F* |
| --- | --- | --- | --- | --- | --- |
| Model | 10954.57 | 5 | 2190.91 | 819.51 | < 0.0001 |
| Error | 13.37 | 5 | 2.67 |  |  |
| Total | 10967.93 | 10 |  |  |  |
| Lack of fit | 12.61 | 3 | 4.20 | 11.08 | 0.0839 |
| Pure error | 0.76 | 2 | 0.39 |  |  |
| Total Error | 13.37 | 5 |  |  |  |

R^2^ = 0.999; adjusted R^2^ = 0.998; root mean square error = 1.635; SS, sum of squares; DF, degrees of freedom; MS, mean square.

**Table S5.** Relative abundance of glycans present in rrhGAA collected from rice cell cultures of control without mannosidase inhibitors and optimal concentration of mannosidase inhibitors (3.84 µM KIF and 8.20 µM SWA) for Man7/8/9 contents

|  | | Control | Optimal concentration |
| --- | --- | --- | --- |
| *N*-glycan structure | | Relative abundance (%)^a^ | |
| Paucimannose type glycan | |  |  |
| MM | 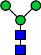 | 2.7 ± 0.0 | 0.4 ± 0.0 |
| MMX | 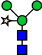 | 7.5 ± 0.0 | 1.1 ± 0.1 |
| MMXF^3^ | 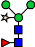 | 23.5 ± 0.1 | 3.4 ± 0.0 |
| Complex type glycan | |  |  |
| MGn | 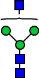 | 3.1 ± 0.2 | - |
| MGnX | 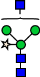 | 5.4 ± 0.4 | 0.2 ± 0.1 |
| GnGn | 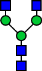 | 1.4 ± 0.4 | - |
| GnGnX | 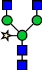 | 2.8 ± 0.3 | - |
| AMX | 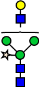 | 0.4 ± 0.1 | - |
| GnMXF^3^ | 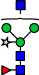 | 9.6 ± 0.0 | - |
| GnGnXF^3^ | 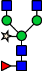 | 33.8 ± 0.1 | 0.8 ± 0.0 |
| AMXF^3^ | 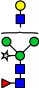 | 2.8 ± 0.0 | - |
| AGnXF^3^ | 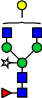 | 2.0 ± 0.1 | - |
| AFGnXF^3^ | 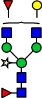 | 4.5 ± 0.1 | - |
| Hybrid type glycan | |  |  |
| Man5XF^3^ | 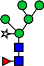 | - | 0.8 ± 0.1 |
| High-mannose type glycan | |  |  |
| Man5 | 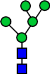 | - | 0.4 ± 0.0 |
| Man6 | 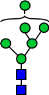 | 0.5 ± 0.1 | 1.4 ± 0.0 |
| Man7 | 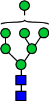 | - | 3.0 ± 0.1 |
| Man8 isomerC | 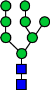 | - | 2.0 ± 0.1 |
| Man8 isomerA | 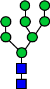 | - | 10.0 ± 0.0 |
| Man8 isomerB | 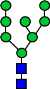 | - | 4.1 ± 0.0 |
| Man9 | 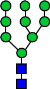 | - | 72.3 ± 0.1 |

^a^Values are the average of triplicates instrument runs from the same preparation.

-, not detected; ■, *N*-acetylglucosamine; ●, mannose; ●, galactose; ▲, fucose; ☆, xylose.

**
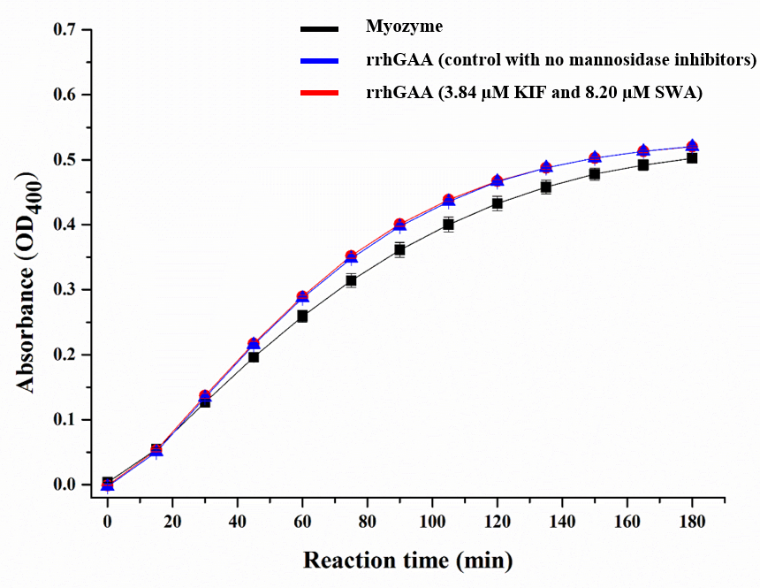
**

**Figure S1.** *In vitro* enzyme activity assay of the purified rhGAAs using *p*-nitrophenyl α-D-glucopyranoside (*p*-NPG) as a substrate.


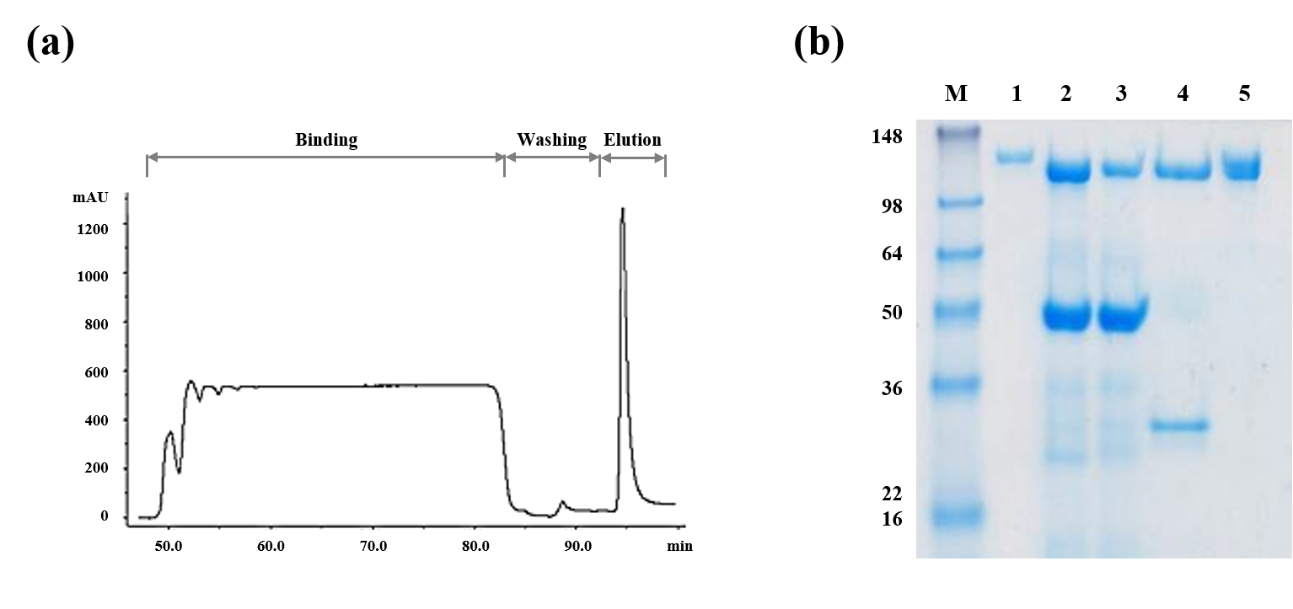


**Figure S2.** One-step purification of rrhGAA using Ni-NTA superflow column. (a) Chromatogram profile of rrhGAA eluted from the Ni-NTA superflow column and AKTA FPLC system (GE Healthcare biosciences, USA). (b) SDS-PAGE analysis of chromatography fractions. M, SeeBlue^®^ Plus2 pre-stained protein marker; 1, 250 ng of Myozyme^®^ (Genzyme); 2, suspension cultured media; 3, unbounded flow-through; 4, washes; 5, eluate

**Supplementary Materials and Methods**

**Enzyme activity of rhGAAs**

Enzyme activities of the purified rhGAAs were measured using the *p*-nitrophenyl-D-α-glucopyranoside (*p*-NPG) (Santa Cruz Biotechnologies, Dallas, TX, USA)^1^. Reaction buffer (10 mM of *p*-NPG, 50 mM sodium acetate and 0.1% BSA; pH 4.3) was pre-warmed to 37°C for 5 min. 5 µg of Myozyme^®^ (Genzyme) as a standard substance and the purified rrhGAAs were loaded in a 96-well microplate (Sigma-Aldrich) and 200 µL/well of reaction buffer was added. Absorbance values at 400 nm of the hydrolyzed *p*-nitrophenol (*p*-NP) were monitored by microplate reader (Thermo Fisher Scientific) at 37°C for 180 min.

**Supplementary References**

1. Jung, J.-W., Kim, N.-S., Jang, S.-H., Shin, Y.-J. & Yang, M.-S. Production and characterization of recombinant human acid α-glucosidase in transgenic rice cell suspension culture. *J. Biotechnol.* 226, 44–53 (2016).
